# A Few Practical Remarks upon Erysipelas of the Head

**Published:** 1842-12

**Authors:** Charles S. Tripler

**Affiliations:** Surgeon U. S. Army; Detroit Barracks


Art. III.—*A few practical Remarks upon Erysipelas of the Head.* By Charles S. Tripler, M. D., Surgeon U. S. Army.

For obvious reasons, inflammation has occupied a prominent place in the writings of medical authors, from the dawn of our science to the present day. The frequency of its occurrence upon the superficial tissues of the body, must of necessity have attracted to it a particular degree of attention. Its phenomena have, hence, been always as accurately observed and recorded as the state of physiological knowledge would admit. So palpable a distinction as that between phlegmon and erysipelas could hardly have escaped attention at any time; accordingly, we find it noticed among the earliest writers. But, from the imperfect knowledge, or rather the entire ignorance, of the ancients, of all just anatomical and physiological notions, we need not be surprised to find that they had no conception of the important varieties of each of these two kinds of inflammation.

To this remark, however, there is one exception, and that is, in the case of erysipelas affecting the head. The particular danger of this variety, was early noticed. Celsus is quite emphatic on the point—"Id autem," says he, "quod erysipelas vocari dixi, non solum vulnere supervenire, sed sine hoc quoque oriri consuevit, atque interdum *periculum majus* affert, utique si circa cervices aut caput constitit."*

But although he here distinctly recognizes the increased danger of erysipelas occurring spontaneously, and especially if affecting the head, he still seems to consider it as merely a variety of erysipelatous inflammation, and not as possessing any specific character. This distinction is comparatively of

* He goes on to prescribe blood-letting, refrigerants, discutients, &c. Among his local remedies he has anticipated the favorite application of Hennen and Liston in local inflammation—"Quicquid impositum est, beta folio contigendum est, et super, linteolum, frigida aqua madens, imponendum."

a modern date and even now is by no means universally, I might say not even generally admitted. I think it an important one, however, in a practical point of view, and I must confess my own opinion is decidedly in favor of its truth.

In the middle of January, 1840, I repaired to Detroit, Michigan, to assume the charge of the Army Hospital at that post. The garrison was then composed of a detachment of the 4th Regiment of Artillery, consisting of about 350 officers and men. Erysipelas of the head had appeared a short time before my arrival, and I found one man convalescent with it in the hospital, and one sick with the disease, who died soon after. Ten more cases occurred during the first quarter of that year. From that time up to last January, sporadic cases have from time to time presented themselves among our men, and I have frequently heard of other cases in the private practice of the medical gentlemen of the city.

Of the first ten cases, six came on with ordinary symptoms of fever, two with cynanche, one with parotitis, and one with pneumonia. The period of the developement of the eruption was from two to four days, and in one case (fatal one) fifteen days. The duration of the disease in the successfully treated cases was from eight to forty-four days. The cause of this affection, as it prevailed at Detroit, I am inclined to think, was the peculiar poison of typhus fever. An eminent authority observes, that "no very exact information has yet been established respecting the causes of erysipelas. We absolutely know nothing about the immediate cause; the prevailing ideas concerning the predisposing causes are vague; and only those causes termed exciting appear entitled to much confidence."

The causes usually enumerated are, external irritants, heat and cold, wounds and other mechanical injuries, intemperance, derangement of the digestive organs and contagion. Most of these causes can only be assigned as producing *accidental* erysipelas, the seat of which will be that part to which the cause was immediately applied. That form of the disease of which we are treating, must arise from a more specific agent. It was suggested to me that the cause of my cases, was the sudden alternations of temperature to which soldiers are subjected when on guard in the winter season—leaving a close guard room, heated to an inordinate degree by stoves, standing post in very cold weather for two hours, and then returning to the hot room. This is not satisfactory; for most of the men of the command were subjected to the same influence in the course of the season, and yet comparatively few became subjects of the disease. And, moreover, one of the cases occurred in a man who had never been so situated; his neatness as a soldier causing him to be invariably selected as orderly to the commanding officer, when detailed for guard. I also frequently inspected the company quarters during the season, and never found the barrack room too much heated, nor imperfectly ventilated.* I suspected at one time, the cause might be found in the unfavorable construction and location of the main barrack—a building put up for a store house, built of wood with low lofts, three stories high, and situated immediately upon the margin of the river, on the lowest ground in the city. But, two companies of the detachment were quartered in another establishment, remote from the river, and on much higher ground. Among these two companies their due proportion of cases occurred. Again, some few cases occurred among the citizens, and one in a lady in the most extensive hotel in Detroit. One or more cases of the disease also occurred at the arsenal, twelve miles from Detroit.

Intemperance cannot be assigned as the cause, for two of the first ten cases occurred in men perfectly temperate, while among the many intemperate men of the command only eight more cases appeared. Intemperance can be considered only as a cause predisposing to disease generally, while the cause of the particular form the disease shall assume, must be sought elsewhere.

There is no field in the wide domain of medical enquiry,

---

* That is, the ventilation was as complete as it could be in such contracted quarters. There were too many men and too few windows for the size of the rooms.

so carelessly cultivated, as that of the etiology of diseases. A disease appears, and an attempt is immediately made rather to *assign* than to *discover* a cause for it. Something suggests itself which seems to satisfy the problem, while, in fact, it may have no relation to it whatever, and the most it should lay claim to, would he a low degree of probability. Nevertheless, it is frequently too readily adopted, and too generally assented to, until another writer shall (as is usual in our profession) first show the inadequacy of the cause assigned by his precursor, and then proceed to attribute the effect to another cause, in all probability equally conjectural. The truth may perhaps be hidden among the multiplicity of errors advanced, but who is able to find it?

Erysipelas of the head has thus been attributed to contagion, and strong facts are adduced in support of this opinion by Dr. Stevenson, Dr. Wells and others. This doctrine, however, can boast few supporters at the present day. It seems to be the fashion now to dispute the contagious nature of all febrile diseases. But the question can hardly be considered as settled. If it were even admitted that a disease were contagious, it by no means follows, in my opinion, that it can be reproduced in no other way. It is evident that the first subject of it could not have derived the poison from contagion. There must have been some peculiar conjunction of appropriate elements that resulted in the production of the poison in the first instance, and I can conceive of nothing that forbids the idea that such conjunction may again occur. Cholera, yellow fever, plague, typhus, &c., I cannot doubt are sometimes contagious, and sometimes of spontaneous origin. The contagionist shows conclusively, to my mind, that these diseases have been propagated by contagion, and if he would stop here, he would do well. But he must go a step further, and argue, that because they are sometimes contagious, they are always so, and can be reproduced in no other way; clearly a *non sequitur*, and he cannot so control facts as to bear him out. His antagonist is no better off; not content with demolishing the position of the contagionist (which he can easily do) he must impale himself on the opposite horn of the dilemma.

After all, it would save a deal of useless labor and controversy, if observers would content themselves with a simple statement of facts as they occur, and when a sufficient number shall have been accumulated, perhaps an intelligible explanation of the origin of particular epidemics may be extracted from them.

But to return to the erysipelas at Detroit. I have expressed my opinion, that it was neither produced by intemperance, alternations of heat and cold, nor by the particular location of the barracks. I may now add that I saw no reason to suppose it contagious *in this instance.* What then was the cause? I do not know—I can only suggest, and that not without hesitation, that it was a form of typhus fever. The London Medico-Chirurgical Review, for July, 1839, contains an analysis of a work on typhus fever by Dr. Roupen. Among the forms which this disease assumed in the London hospitals, he enumerates erysipelas. "During the prevalence of the epidemic (typhus) in various years, idiopathic erysipelas has been a very common and a very serious addition to other complaints in persons exposed from their situation in hospitals and elsewhere to the vicinity and infection of typhus, and so common was it in the progress of the fever during the present year as well as during that of 1831, that no doubt could be entertained that it was essentially connected with and incident to this disorder." "It prevails at the same time as typhus, is preceded by the same symptoms and arises amongst nurses or those in attendance upon the patients ill with that fever."

It is a well ascertained and an admitted fact, that typhus, though undoubtedly "a disease of the whole system", still impinges more powerfully upon some one or more distant organs; and it would even appear, that in particular endemics or in particular districts, the disease seems almost invariably to seize upon the same organs to the entire exclusion of the rest. If this be true, it may account in a great degree for the diversity of opinion among writers as to the seat of the disease—one placing it in the brain, another in the stomach, a third in the glands of Peyer and so on, while all are sometimes true, and none exclusively so. Now, why may not typhus sometimes expend its force upon the skin? What should give the poison that particular direction, I do not know; nor do I know why it should sometimes select the lungs or brain for its point of attack. The reason why some one or more of the glandular apparatus* is selected, I suppose to be the exalted vascularity of those tissues; and in this respect as well as others the dermoid tissue may be classed among them. If the pulmonary tissue were extended over a surface equal to that of the dermoid, I apprehend erysipelas would be as appropriate a term for the pneumonia of typhus as it is now for the cutaneous disease.†

Samuel Cooper remarks that, "Every surgeon is well aware that one cause of erysipelatous inflammation is a fever of a determinate and peculiar nature, one feature of which is, the invariable production of this kind of inflammation upon the surface of the body." If he had said, one cause is the poison of typhus under peculiar circumstances as yet unknown, he would have conveyed a practical, intelligible and just idea of this particular form of erysipelas.‡

The supposition, then, that the disease as it prevailed here, was a form of typhus, seems to me at least to be a plausible one. It occurred under the circumstances in which we ordinarily expect to find typhus; about the same proportion of persons exposed were attacked as in moderate typhus endemics, and the symptoms, exclusive of the eruption, were very similar to those of typhus. It should be stated, there were no cases of the usual form of typhus during the prevalence of the erysipelas.‖

* I have not used this term in its strict, but in a free sense.

† See Hunter on Inflammation.

‡ Erysipelas may also be one of the phenomena of an intermittent or any other form of fever.

‖ These remarks refer to the barracks only.

Dr. Good places erysipelas among his exanthemata. He has divided the general subject, with a view of drawing a line of demarkation between erythema and erysipelas proper. He considers the fever in erythema as only symptomatic, and the eruption the primary disease; while in erysipelas this order of cause and effect is inverted. Whatever may be the justness of his views with regard to erythema, I fully coincide with them in reference to erysipelas of the head. Mr. Lawrence disputes the propriety of placing erysipelas among the exanthemata, because it is often not preceded by fever,* because it is doubtful whether it is contagious, and because it attacks the same individual repeatedly. This is evidently taking a limited rather than an enlarged, a surgical rather than a philosophical view of the matter. No one pretends that every case of erysipelas is preceded by fever, but no one who has been favored with opportunities for observation, can doubt, that a particular form of fever may prevail, one of the phenomena of which will, in the generality of cases, be erysipelas. Now, if from effects we can sometimes infer causes, and if identity of both cause and effect be necessary to establish identity of *essence,* we may conclude that the disease under discussion is *not* the same either in cause or effect as the erythema of Good or most of the forms of the erysipelas of Lawrence; nor has sufficient yet been established by observation, to determine whether it ought, or ought not, to be classed among the exanthemata, even under the conditions prescribed by Mr. Lawrence. This is an important practical matter, and Mr. Lawrence seems to be in error in supposing, if I understand him right, the causes of erysipelas to be exclusively those of phlegmon, and the effect, merely a particular modification of cutaneous or cutaneous and cellular inflammation. Every case of erysipelas is not a case of typhus, nor is every pneumonia; but that both are sometimes symptoms or phenomena of typhus, I cannot doubt; and this is the mistake of Lawrence and others, in confounding the specific affection with the

* This will depend altogether on the *cause* of the disease.

erythema of Good or with erysipelas arising from extreme irritants, mechanical injuries, &c.

Burserius divides the disease into idiopathic, symptomatic and accidental. This division I should decidedly prefer to that of Mr. Lawrence, into erythema, simple, œdematous and phlegmonous erysipelas. I doubt whether an endemic erysipelas of the head ever prevailed, without exhibiting instances of all Mr. Lawrence's species, as symptoms of what I believe to be the true disease—typhus fever.

Mackintosh denies "altogether the idiopathathic nature of erysipelas, and believes it to be an occasional symptom of different diseases, which diseases may frequently occur under atmospheric, epidemical, and contagious influences." This general remark seems to cover the ground taken by Roupen and to sustain the opinion that it is sometimes a symptom of typhus. But I should judge from what follows in his (Mackintosh's) article on the subject, that he had excluded in his own mind the idea of typhus when he wrote the paragraph which I have quoted. He evidently considers it as an active inflammation, and requiring always the free use of the lancet. Now, I am far from disputing that such may have been the case in Edinburgh during his experience, but I am very confident that in other places its nature is sometimes very different, I cannot agree with him either, in his notion that the eruption ought to be considered a natural blister; at least in epidemic erysipelas. In my cases the constitutional disturbance did *not* subside as the eruption appeared.

Mackintosh goes on to quote Cullen's history of the symptoms of erysipelas of the head. That history is a very good history of the accession of inflammatory typhus. He then quotes Sydenham's letter on the plague of 1655, and from this it appears that that epidemic occasionally assumed the form of erysipelas. All these things serve, in my opinion, to corroborate the notion that the disease is a variety of typhus. As to Mr. Abernethy's saying that, "he would be hanged if erysipelas was not *always* the result of a disordered state of the digestive organs," it merely proves, that though a clever physician, that gentlemen was no prophet. Hippocrates and Galen before him, had assigned it a similar origin, and that it does sometimes arise from disorder of the digestive apparatus, there can be no doubt; but "*always*," is quite another matter.

I have dwelt more at length on this part of my subject, that I may be permitted to suggest to such of my professional brethren as shall take the trouble to read this paper, and who may be so situated as not to be likely to see cases of the disease in numbers (as in our hospitals), to be on their guard when they meet with a sporadic case, and not to decide too hastily that it is a mere inflammation. I assume it as a fact that sporadic cases of typhus are usually much more dangerous than cases occurring during an epidemic, and hence the first steps in the treatment are of great importance. I shall not trouble my readers with a general description of the disease, but shall content myself with quoting a single case, which will show the usual source and character of the disease as it presented itself to my observation at this post.

H——, a private of the 4th Regiment of Artillery, reported sick 14th March, 1840, with sore throat. He is intemperate and has been drinking lately. An emetic was prescribed and tinct. ammon. to be applied externally. 15th. Symptoms the same; sol. antimon. tart, ordered every two hours. 16th. Same; the inflammation is a diffused scarlet redness, with slight ulceration of the tonsils, but without any particular tumefaction; continued sol. ant.; sol. nit. argent. to the throat. Evening—blister to the throat. 17th. The throat is better, but during the last night erysipelas made its appearance on the cheek and nose, which spots had joined in the morning. Bowels open; tongue moist, with a slight yellowish fur; no headache; pulse natural; skin dry, and a little hotter than natural. Tr. iodin. to the face; cont. nit. argent. to the throat; cal. gr. iij, p. Doveri gr. v. every three hours. Evening—more excitement; tongue dry; inflammation and tumefaction extending to the other side of the nose. Continued cal. and p. Dov.; applied sol. mur. hyd. to face. 18th. Inflammation extends to the whole of the right side of the face, including the parotid, eye, and eyelids, and slightly affects the left side. The sol. mur. hyd. has already cauterised the oldest inflamed spots. The pulse is full, soft, equable, but somewhat quickened; tongue moister than last night, less furred, but shrivelled; bowels open. Continued remedies. Evening—swelling extends to the scalp and left cheek; pulse soft, full, and rather frequent; skin moist; bowels not moved to-day; mind clear; continued remedies. 19th. Tumefaction same as last night; slept well; bowels not moved; pulse 100; skin soft, but hotter and dryer than natural; tongue dry, wrinkled, with brownish mucus on the sides. Enema; continue cal., p. Dov. and sol. mur. hyd. to the face. 20th. Slight febrile heat; tongue dry and brownish; thirst increased; bowels open; stools black; pulse soft, 104; no delirium; no local pain; slept well, parotid rather more swollen; cuticle begins to shrivel and detach itself; ordered sol. bicarb. potas. 21st. Five dejections since last evening, lighter in color and thin; slept well when not disturbed by the bowels; no delirium; tumefaction subsiding; tongue dry and chapped, but not glazed; pulse 92, soft; and of sufficient volume; skin soft and dry; temperature slightly elevated; yeast poultice to face; continued sol. potas. 22d. Rested well; skin natural; pulse 88; tongue becoming softer at the edges, still shrivelled and wasted in the middle; bowels open; stools becoming lighter and more consistent; cuticle detached over the whole inflamed surface; pus about the eyes, as in small-pox; inflammation of the eyes does not affect the cornea. 23d. Skin dry; temperature natural; pulse weak, 80; tongue dry, brown, and shrivelled in the centre, moist and red at the edges; some slight indications of typhomania exhibited in a desire to get up and sit by the window; bowels twice moved in the night; stools thin and yellow; infus. serpentariæ every three hours. 24th. Skin natural; pulse 84; tongue moister and cleaning off; inflammation of the eyes has subsided; desquamation of the cuticle going on; bowels open; stools light colored and of tolerable consistence; slept well last night; continue serpentaria. 25th. Pulse 72; skin cool; tongue soft and moist; slight mercurial fetor of the breath; bowels open; desquamation of the cuticle progressing; tumefaction gone, except on the anterior portion of the right parotid, which threatens an abscess; continue serpentaria. 26th. Skin natural; pulse 68; tongue moist and almost clean; slept well; two stools since last night, improving in character; slight degree of deafness; inclined to doze. 27th. Still improving; parotid proceeding to suppuration. Patient out of danger.

The case above cited, I have presented as exhibiting the phenomena of the disease as it occurred in Detroit, in its simplest form. I quote it as a sample of the features of the inflammation, rather than with a view to determine from it the nature of the fever. Many cases were much more severe as to the constitutional symptoms; the local disease seldom or never extended farther. In the severer cases, there was more delirium, more prostration; the stage of collapse was more threatening, demanding the exhibition of more powerful stimulants. There was no sloughing of the cellular tissue, and I believe *that* is not an ordinary occurrence in this form of erysipelas. After the subsidence of the specific inflammation, small local abscesses occasionally occurred on different parts of the head, sometimes on the scalp, in one case in the cellular tissue of the eyelids, and in the case cited, in the parotid region. Swelling of the parotid was a common occurrence. In a large proportion of the cases, deafness supervened, as the erysipelas subsided. Both deafness and local abscess about the head, have frequently occurred in cases of typhoid fever that have fallen under my observation.

Upon a review of the phenomena of this disease, and the circumstances under which it occurred, both during the period which more particularly suggested these observations and since that period, I think it ought to be considered as a form of typhus, and that its appearance should be attributed to the presence of the poison, whatever it may be, that produces that disease. From the opinion I have expressed as to the *nature* of the disease, it will readily be inferred that I consider a proper *constitutional* treatment of paramount importance. And herein do I differ most widely from those who treat of erysipelas as a modification merely of inflammation. But without occupying time or space in recapitulating the treatment of others, I shall proceed to give my own in as few words as possible, merely premising, that whether right or wrong in my notions of the pathology of the disease, my treatment has been attended with gratifying success.

Blood-letting, at the onset, was of singular service. Though I never saw it arrest the disease at once, as Mackintosh says it frequently does, still it has evidently rendered the inflammation milder, and above all, has checked the general morbid action. Whether it would be as beneficial in future epidemics, is a matter of uncertainty. There can be no doubt that the epidemic meteoration of different seasons decides the character of those seasons, so that it requires renewed observation in each season to determine the applicability of this as well as other heroic remedies. The general character of the diseases which prevailed at Detroit during the early part of 1840, was inflammatory. Blood-letting was always borne well, not only in my practice, but in that of the medical gentlemen in the city. Even in the habitually intemperate, it answered well when they were subjects of erysipelas.

An early exhibition of emetics and purgatives, or both, was always resorted to, on the accession of the disease. After these things had been premised, the permanent constitutional and local treatment was at once adopted and persevered in. The former consisted of calomel and p. Doveri, in small and frequently repeated doses, during the stage of excitement, with infusion of serpentaria, wine whey, carb. ammonia, &c., during the stage of collapse; the latter, of a strong solution of the deuto-chloride of mercury.

It is not necessary to say any thing more of the *general* means, but the *local remedy* has proven so eminently successful, that I cannot omit to recommend it strongly to the notice of the profession.

I had made trial of the usual evaporating lotions, the mercurial ointment, the sol. nit. argent., &c., without any satisfactory result. The nitrate of silver I consider the best among them, but it does not produce the immediate and powerful impression that follows the use of the corrosive sublimate. This remedy was suggested to me by Dr. Pitcher, of Detroit (formerly of the army,) who had received it from his preceptor. I have never seen it recommended in books. Dr. Pitcher told me, that his preceptor thought it not only arrested the local disease, but also protected the brain. Of the latter I have no doubt, though I think it does not prevent the inflammation from extending pretty generally over the face and head. If any of my readers should be induced to try the remedy, I warn them not to expect the spread of the inflammation to be forthwith arrested by it; if they do, they will be disappointed. I was in the habit of applying it always a little beyond the bounds of the inflammation. It produced no effect upon the sound skin, but on the diseased integument, its effect was rapid and decisive, twenty-four hours being sufficient to cauterize the cuticle completely. It must have acted as a powerful counter-irritant in relation to the brain, for after its application, notwithstanding the apparent severity of the local disease, the brain rarely, and even then but slightly, suffered. When delirium occurred it was always moderate and never much protracted. It was remarkable, that during that winter all the cases treated with the corrosive sublimate, as far as I know, recovered; while two, to my knowledge, treated without it, died. Since then I have witnessed two unsuccessful cases; but the entire ruin of the constitution in those cases from intemperance and all sorts of vice, was sufficient to account for the disastrous result. I therefore look upon this remedy as an important means in the treatment of erysipelas of the head. The solution I used was composed of 20 grains of the deutochloride of mercury to one fluid ounce of water. A friend of mine in the city used a saturated solution in a frightful case, successfully. It is applied with a pencil or piece of lint over the inflamed surface and a little beyond, four or five times in the course of a day, taking care to guard the eyes, nostrils and mouth. During the prevalence of the complaint, I met with some observations by Mr. John Davies of Hertford, England on the use of iodine in various local diseases. Among others, he recommends it strongly as a topical application in erysipelas. I made trial of it according to his plan in two cases. One was very mild, with the slightest degree of constitutional disturbance, and the local involving merely one side of the nose and a small portion of the cheek. In this case it was successful. The other case I have recorded above. It did not seem, in this instance, to produce the least effect during the short time I used it, and as the symptoms were alarming, I did not feel justified in relying upon an untried remedy, while I possessed one that experience had shown to be worthy of dependance. I therefore fell back upon the corrosive sublimate. I do not profess to have given the tr. iodine a decisive trial, but I must confess, from my experiment, I should not place much reliance upon it, and I consider it far inferior to the other remedy.

Detroit Barracks, Nov. 1842.

